# Multiple Neuritis in a Child Due to Lead Poisoning

**Published:** 1905-07-08

**Authors:** 


### MULTIPLE NEURITIS IN A CHILD DUE TO LEAD POISONING.

A VERY interesting case of nervous disease in a child and due to the action of lead is recorded by Dr. Robt. Hutchison.[1] The patient was a boy of six years who suddenly lost the use of his legs, the onset of the paralysis being accompanied by vomiting and slight fever. Examination showed a flaccid paralysis of the anterior tibial muscles on each side. The diagnosis offered was one of anterior polio-myelitis, and this seemed to be confirmed by subsequent contracture of the calf muscles and the development of talipes equinus. But some fifteen months after the first attack the boy suddenly became paralysed in the upper limbs and the muscles of the trunk, including the diaphragm. This necessarily demanded a reconsideration of the diagnosis as an anterior polio-myelitis of such an extent could not be regarded as possible. The case was, therefore, now viewed as a multiple neuritis of motor type, the sensory functions being undisturbed. In the endeavour to determine the cause of the neuritis a blue line was discovered on the gums, and it was found that the patient had acquired the habit of earth-eating and of sucking the paint off his toys. From this source it was concluded that the lead-poisoning took origin. Doubtless, in view of these later facts, it must be recognised that the first attack was due to peripheral neuritis and not to a central lesion. The peroneal form of lead-poisoning is uncommon, whilst a multiple toxic neuritis due to lead is excessively rare.

[1] The Polyclinic Jour., July 1905.

